# Supplementary material for: Identification of Freezing-Responsive microRNAs and Their Targets in Chinese Jujube by Small RNA and Degradome Sequencing
Source: Int J Mol Sci. 2026 Sep 4;27(17):7912. doi: 10.3390/ijms27177912 (PMC13566890; doi:10.3390/ijms27177912)
Supplement: Supplementary file 1 [file ijms-27-07912-s001.zip › Supplementary Material S2/novel_miR_19.pdf]

Provisional ID	:	novel_miR_19
Score total	:	6070.7
Score for star read(s)	:	3.9
Score for read counts	:	6061.4
Score for mfe	:	3.8
Score for randfold	:	1.6
Score for cons. seed	:	
Total read count	:	11901
Mature read count	:	11808
Loop read count	:	92
Star read count	:	1

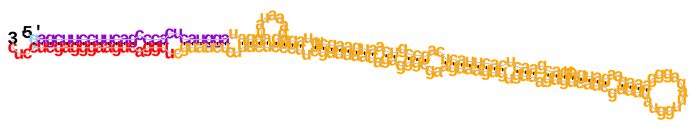

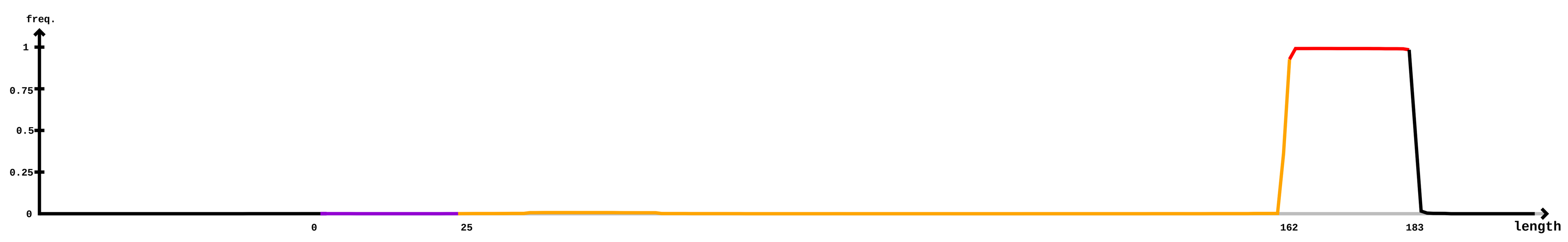

Star **Mature**

```
5'- agguaauccaauggauaccaugaguggaagaguaauggaaagugagagcuuccuucagcccacucaugga uaggauauaaaggggaugucgaaguaacugccgacucauucacucaagcacucaguagaagaaagggagauugguauuuugcuacugugauugugugaaugaagcgggagauauuuugcuucccucuuucucuaugcuuggacugaagggagcuccuccuuuucauuugccucuauuauu -3' obs
    agguaauccaauggauaccaugaguggaagaguaauggaaagugagagcuuccuucagcccacucauggauaggauauaaaggggaugucgaaguaacugccgacucauucacucaagcacucaguagaagaaagggagauugguauuuugcuacugugauugugugaaugaagcgggagauauuuugcuucccucuuucucuaugcuuggacugaagggagcuccuccuuuucauuugccucuauuauu exp
    .((((.(((...)))))))...((((((.(.(((((((((((.((((((((((((((.(((..((((((.((((......((((.(.(((((((.((.(((..((((((((.(((.(((..(((((..((((...........)))).))))))))).))).))))))))..))).)).)))))))).))))))))..))))))..))).))))))))))))).))))))))))).)))))))))))...
```

read	reads	mm	sample
...aauggaaagugagagcuu...	4	0	JT-1
...ugagagcuuccuucagcccacu...	1	0	LT-3
...aNcuuccuucagcccacucaugga...	1	1	JCK-3
...uggauaggauauaaaggggaugucgaagu...	1	0	LT-2
...uggauaggauauaaaggggaugucgaagu...	1	0	JT-1
...uggauaggauauaaaggggaugucgaagu...	2	0	JCK-2
...gauaggauauaaaggggaug...	1	0	LT-1
...uaggauauaaaggggaugucgaag...	1	0	LT-3
...uaggauauaaaggggaugucgaagu...	1	0	JCK-2
...uaggauauaaaggggaugucgaagu...	1	0	LT-3
...uaggauauaaaggggaugucgaagu...	2	0	JCK-3
...aggauauaaaggggaugucgaagu...	2	0	JCK-2
...aggauauaaaggggaugucgaagu...	1	0	LCK-3
...uauaaaggggaugucgaagua...	1	0	JCK-2
...uauaaaggggaugucgaagua...	1	0	JCK-3
...uauaaaggggaugucgaaguaacugcc...	1	0	LCK-3
...auaaaggggaugucgaaguaacu...	1	0	JCK-3
...uaaaggggaugucgaaguaac...	1	0	LT-2
...uaaaggggaugucgaaguaacugcc...	2	0	JCK-2
...uaaaggggaugucgaaguaacugcc...	1	0	JCK-3
...aggggaugucgaaguaacugcc...	6	0	JT-1
...aggggaugucgaaguaacugcc...	2	0	LT-2
...aggggaugucgaaguaacugcc...	6	0	JT-2
...aggggaugucgaaguaacugcc...	15	0	JT-3
...aggggaugucgaaguaacugcc...	2	0	LT-1
...aggggaugucgaaguaacugcc...	2	0	JCK-3
...aggggaugucgaaguaacugcc...	5	0	LT-3
...aggggaugucgaaguaacugcc...	14	0	LCK-3
...aggggaugucgaaguaacugccg...	2	0	LT-2
...gggaugucgaaguaacugccgacuc...	3	0	JCK-1
...gggaugucgaaguaacugccgacuc...	2	0	JCK-2
...ggaugucgaaguaacugccgacuc...	1	0	JCK-3
...aacugccgacucaNucacu...	1	1	LCK-1

Star Mature

```
agguaauccaauggauaccaugaguggaagaguaauggaaagugagagcuuccuucagcccacucaugguaggauauaaaggggaugucgaaguaacugccgacucauucacucaagcacucaguagaagaaagggagauugguauuuugcuacugugauugugugaaugaagcgggagauauuuugcuucccucuuucucuaugcuuggacugaagggagcuccuccuuucauuugccucuauuauu
...............................................................................................aNcugccgacucauucacuca.....................................................................................................................................  1    1  LT-1
...............................................................................................aacugccgNcucauucacuca.....................................................................................................................................  1    1  JCK-3
...............................................................................................aaNugccgacucauucacuca.....................................................................................................................................  2    1  LT-2
...............................................................................................aNcugccgacucauucacuca.....................................................................................................................................  1    1  JCK-3
...........................................................................................................auucacucaagcacucaguagaagaa....................................................................................................................  1    0  LCK-3
...................................................................................................................................aaagggagauugguauuuug..................................................................................................  1    0  LT-3
...................................................................................................................................................................ugugaaugaagcgggagauauuuugcu...........................................................  1    0  JT-1
........................................................................................................................................................................................uuugcuucccucuuucucuaug...........................................  1    0  JCK-2
........................................................................................................................................................................................uuugcuucccucuuucucuaug...........................................  1    0  JCK-3
.................................................................................................................................................................................................cucuuucucuaugcuuggac....................................  1    0  LT-1
..................................................................................................................................................................................................ucuuucucuaugcuuggacug..................................  1    0  JCK-3
..................................................................................................................................................................................................ucuuucucuaugcuuggacug..................................  3    0  JCK-1
.........................................................................................................................................................................................................cuaugcuuggCcugaaggga............................  1    1  JCK-2
.........................................................................................................................................................................................................cuaugcuuggacugaagggU............................  2    1  LT-1
.........................................................................................................................................................................................................cuaugcuuggacugaagggagU..........................  2    1  JCK-2
.........................................................................................................................................................................................................cuaugcuuggacugaagggagU..........................  3    1  LT-1
.........................................................................................................................................................................................................cuaugcuuggacugaagggagU..........................  1    1  JCK-3
.........................................................................................................................................................................................................cuaugcuuggacugaagggagU..........................  1    1  LT-3
..........................................................................................................................................................................................................uaugcuuggCcugaagggagcucc.......................  1    1  JCK-1
............................................................................................................................................................................................................ugcuuggacugaagggagcucUuc.....................  1    1  LT-1
.............................................................................................................................................................................................................gcuuggacugaagggagcuccucU....................  1    1  LT-1
..............................................................................................................................................................................................................cuuggacugaagggagcucAu......................  1    1  LT-1
..............................................................................................................................................................................................................cuuggaUugaagggagcuccu......................  4    1  JCK-2
..............................................................................................................................................................................................................cuuggacugaagggagcucAu......................  1    1  JCK-3
..............................................................................................................................................................................................................cuuggCcugaagggagcuccu......................  1    1  JT-3
..............................................................................................................................................................................................................cuNggacugaagggagcuccu......................  2    1  LT-2
..............................................................................................................................................................................................................cuuggCcugaagggagcuccu......................  1    1  LCK-3
..............................................................................................................................................................................................................cuuggacugaagggCgcuccu......................  1    1  JCK-3
..............................................................................................................................................................................................................cuuggaUugaagggagcuccu......................  12   1  LT-3
..............................................................................................................................................................................................................cuuggacugaagggagcCccu......................  1    1  JCK-3
..............................................................................................................................................................................................................cuuggaUugaagggagcuccu......................  3    1  JCK-1
..............................................................................................................................................................................................................cuuggacugaagggagcucAu......................  1    1  LT-3
..............................................................................................................................................................................................................cuuggaUugaagggagcuccu......................  5    1  LT-1
..............................................................................................................................................................................................................cuuggacugaagggagcucAu......................  1    1  JCK-1
..............................................................................................................................................................................................................cuuggacugaagggagcucUu......................  1    1  JCK-1
..............................................................................................................................................................................................................cuuggaUugaagggagcuccu......................  3    1  JCK-3
..............................................................................................................................................................................................................cuuggaAugaagggagcuccu......................  1    1  JCK-1
..............................................................................................................................................................................................................cuuggCcugaagggagcuccu......................  1    1  JCK-2
..............................................................................................................................................................................................................cNuggacugaagggagcuccu......................  1    1  JCK-3
..............................................................................................................................................................................................................cuuggacugaagggagcuUcu......................  1    1  LT-1
..............................................................................................................................................................................................................cuugAacugaagggagcuccu......................  1    1  JCK-2
..............................................................................................................................................................................................................cuuggacugCagggagcuccu......................  1    1  LT-2
..............................................................................................................................................................................................................cuuggacugaagggagcucUuc.....................  10   1  JCK-3
..............................................................................................................................................................................................................cuuggacugaCgggagcuccuc.....................  2    1  LT-1
..............................................................................................................................................................................................................cuuggacugaagggagcucNuc.....................  2    1  JCK-1
..............................................................................................................................................................................................................cuuggacugaaggNagcuccuc.....................  1    1  JT-2
..............................................................................................................................................................................................................cuuggCcugaagggagcuccuc.....................  1    1  LCK-3
..............................................................................................................................................................................................................cuuggacugCagggagcuccuc.....................  1    1  JCK-1
..............................................................................................................................................................................................................cuuggacugaagggagcuccuc.....................  820  0  JCK-3
..............................................................................................................................................................................................................cuuggacugaagggagcuccuc.....................  201  0  LT-2
..............................................................................................................................................................................................................cuCggacugaagggagcuccuc.....................  1    1  LT-3
..............................................................................................................................................................................................................cuuggacugaagggagcuccuc.....................  646  0  JCK-1
..............................................................................................................................................................................................................cuuggacugaagggagcuccuc.....................  58   0  JT-3
..............................................................................................................................................................................................................cuuggaAugaagggagcuccuc.....................  2    1  LT-1
..............................................................................................................................................................................................................cuuggacugaagggagcuccuc.....................  157  0  LCK-3
..............................................................................................................................................................................................................cuuggacugCagggagcuccuc.....................  2    1  JCK-2
..............................................................................................................................................................................................................cuuggCcugaagggagcuccuc.....................  1    1  LT-1
..............................................................................................................................................................................................................cNuggacugaagggagcuccuc.....................  1    1  JCK-3
..............................................................................................................................................................................................................cuuggCcugaagggagcuccuc.....................  1    1  JT-2
..............................................................................................................................................................................................................cuuggacugaCgggagcuccuc.....................  1    1  JCK-2
..............................................................................................................................................................................................................cuuggacugaagggagcuUcuc.....................  2    1  JCK-1
..............................................................................................................................................................................................................cuuggacugGagggagcuccuc.....................  1    1  JCK-2
..............................................................................................................................................................................................................cuuggacugaaNggagcuccuc.....................  3    1  JCK-1
..............................................................................................................................................................................................................cuuggacugaagggagcucUuc.....................  1    1  LT-1
..............................................................................................................................................................................................................cuuggacugaagggagcuccuc.....................  399  0  LT-1
..............................................................................................................................................................................................................cuuggacugaagggagcuccuc.....................  8    0  JT-1
..............................................................................................................................................................................................................cuuggacugaagggagcuccuc.....................  64   0  JT-2
..............................................................................................................................................................................................................cuuggacugaagggagcucUuc.....................  15   1  JCK-2
..............................................................................................................................................................................................................cuuggacugaagggagcuUcuc.....................  2    1  JCK-2
..............................................................................................................................................................................................................cuuggacugCagggagcuccuc.....................  2    1  LT-3
```

Star **Mature**

agguaauccaauggauaccaugaguggaagaguaauggaaagugagagcuuccuucagcccacucaugga uaggauauaaaggggaugucgaaguaacugccgacucauucacucaagcacucaguagaagaaagggagauugguauuuugcuacugugauugugugaaugaagcgggagauauuuugcuucccucuuucucuaugcuuggacugaagggagcuccuccuuuucauuugccucuauuauu

cuuggCcugaagggagcuccuc	2	1	JCK-2
cuuggacugaagggagcuccuN	1	1	LT-2
cuuAgacugaagggagcuccuc	1	1	LT-3
cuuggacugCagggagcuccuc	1	1	LT-2
cuuggacugaagggagcuccuc	311	0	LT-3
cuuggacugCagggagcuccuc	1	1	LT-1
cuuggacugaagggagcucUuc	15	1	JCK-1
cuuggCcugaagggagcuccuc	4	1	LT-3
cuuggacugaagggagcuccuc	1048	0	JCK-2
cuuggCcugaagggagcuccuc	1	1	LT-2
cuuggacugaCgggagcuccuc	1	1	JCK-3
cNuggacugaagggagcuccuc	1	1	JCK-1
cuuggCcugaagggagcuccuc	2	1	JCK-3
Uuuggacugaagggagcuccuc	3	1	JT-2
Uuuggacugaagggagcuccuc	8	1	JT-3
cuuggacugaagggagcuccucc	14	0	JCK-1
cuuggacugaagggagcuccuUc	8	1	LT-3
cuuggacugaagggagcuccucU	4	1	JCK-1
cuuggacugaagggagcuccucc	27	0	LT-1
cuuggacugaagggagcuccucc	36	0	LT-2
cuuggacugaagggagcuccucU	5	1	LCK-3
cCuggacugaagggagcuccucc	1	1	LT-3
cuuggacugaagggagcuccucc	20	0	JCK-3
cuuggacugaagggagcuccucc	22	0	JCK-2
cuuggacugaagggagcuccucU	4	1	JCK-2
cuuggacugaagggagcuccucc	27	0	LT-3
cuuggacugaagggagcuccuUc	27	1	JCK-3
cuuggacugaagggagcuccucU	4	1	LT-2
cuuggacugaagggagcuccucU	10	1	LT-3
cuuggacugaagggagcuccucc	6	0	LCK-3
cuuggacugaagggagcuccCcc	2	1	LT-1
cuuggacugaagggagcuccucc	11	0	JT-3
cuuggacugaagggagcuccucA	1	1	LT-1
cuuggacugaagggagcuccucU	8	1	LT-1
cuuggacugaagggagcuccucA	1	1	JCK-1
cuuggacugaagggagcuccuUc	37	1	JCK-2
cuuggacugaagggagcuccucU	3	1	JCK-3
cuuggacugaagggagcuccuUc	5	1	JT-2
cuuggacugaagggagcuccuUc	18	1	JCK-1
cuuggacugaagggagcuccuUc	3	1	LCK-3
cuuggacugaagggagcuccuUc	3	1	JT-3
cuuggacugaagggagcuccuUc	5	1	LT-1
cuuggacugaagggagcuccucA	2	1	JCK-3
cuuggacugaagggagcuccucc	11	0	JT-2
cuuggacugaagggagcuccucA	3	1	JCK-2
cuuggacugaagggagcuccuccu	5	0	LT-2
cuuggacugaagggagcuccucUu	2	1	LT-1
cuuggacugaagggagcuccuccA	18	1	JT-1
cuuggacugaagggagcuccuccA	3	1	JCK-2
cuuggacugaagggagcuccucUu	1	1	LT-3
cuuggacugaagggagcuccuccu	6	0	JT-3
cuuggacugaagggagcuccuccu	6	0	JT-2
cuuggacugaagggagcuccuccA	1	1	LT-2
cuuggacugaagggagcuccuccu	9	0	LCK-3
cuuggacugaagggagcuccuccu	8	0	LT-1
cuuggacugaagggagcuccuccA	4	1	LCK-3
cuuggacugaagggagcuccuccu	8	0	JCK-2
cuuggacugaagggagcuccuccu	6	0	LT-3
cuuggacugaagggagcuccuccu	12	0	JCK-3
cuuggacugaagggagcuccuccu	8	0	JCK-1
cuuggacugaagggagcuccuccA	1	1	LT-3
cuuggacugaagggagcuccuccuu	1	0	JCK-3
cuuggacugaagggagcuccucUuu	1	1	JCK-2
cuuggacugaagggagcuccuccGu	1	1	JCK-1
cuuggacugaagggagcuccucUuu	1	1	LT-1
cuuggacugaagggagcuccucUuu	1	1	LT-3
cuuggacugaagggagcuccucUuu	2	1	JCK-1
cuuggacugaagggagcuccuccuu	1	0	JCK-2
cuuggacugaagggagcuccucUuu	1	1	LT-2
cuuggacugaagggagcuccuccuuuA	2	1	JCK-2

Star **Mature**

agguaauccaauggauaccaugaguggaagaguaauggaaagugagagcuuccuucagcccacucauggauaggauauaaaggggaugucgaaguaacugccgacucauucacucaagcacucaguagaagaaagggagauugguauuuugcuacugugauugugugaaugaagcgggagauauuuugcuucccucuuucucuaugcuuggacugaagggagcuccuccuuuucauuugccucuauuau

cuuggacugaagggagcuccuccuuuuc	3	0	JCK-2
cuuggacugaagggagcuccuccuuuuc	7	0	JT-1
cuuggacugaagggagcuccuccuuuuc	1	0	JCK-1
uNggacugaagggagcuc	1	1	JCK-1
uugNacugaagggagcucc	1	1	JCK-1
uuggaNugaagggagcucc	1	1	LCK-1
uuNgacugaagggagcucc	4	1	LT-2
uuggacugaagggagcucUu	2	1	JCK-3
uuggacugCagggagcuccu	1	1	LT-2
uuggCcugaagggagcuccu	2	1	JCK-1
uNggacugaagggagcuccu	2	1	JCK-2
uuggacugaagggagcuUcu	2	1	LCK-3
uuggacugaagggagcucAu	1	1	JT-3
uuggacugaagggagcucUu	2	1	JCK-1
uuggacugaagggagcucUu	1	1	JCK-2
uuggacugaagggagcucUu	1	1	LT-1
uuggaUugaagggagcuccu	1	1	LT-3
uuNgacugaagggagcuccu	1	1	LT-2
uuggacugaagggCgcuccu	1	1	LCK-3
uuggacugaagggagcucUu	2	1	LT-3
uNggacugaagggagcuccu	1	1	JCK-1
uuggacugaagggCgcuccu	1	1	LT-1
uuggacugaagggagcuccN	2	1	JCK-1
uuggacugaagggagcucAu	1	1	LT-2
uuggacugaCgggagcuccuc	2	1	JCK-3
uuggacugaagggagcucUuc	23	1	JCK-2
uuggacugaagNgagcuccuc	2	1	JCK-1
uuggacugaagggagcuUcuc	3	1	JCK-3
uuggacugaagggagcuccuc	2	0	LCK-2
uuggacugCagggagcuccuc	1	1	JCK-3
uuggacugaCgggagcuccuc	1	1	LT-1
uuggacugaagggCgcuccuc	1	1	JCK-1
uuggacugaagggagcucUuc	4	1	LT-3
uuggacugaagggagcCccuc	1	1	LT-1
uNggacugaagggagcuccuc	4	1	JCK-2
uuggacugCagggagcuccuc	1	1	LT-3
uuggacugaagggagcuccuc	815	0	LT-3
uuggacugaagggagcuccuc	108	0	JT-3
uuggacugaagggagcucUuc	14	1	JCK-1
uuggacugaagggagcuccuc	1024	0	JCK-3
uuggacuAaagggagcuccuc	1	1	LT-2
uuggacugaagggCgcuccuc	1	1	JT-1
uuggacugaagggagcuccuc	842	0	JCK-1
Cuggacugaagggagcuccuc	1	1	JCK-2
uuggacugaagggagcGccuc	2	1	JCK-1
uuggacugaagggagcucUuc	1	1	LCK-3
uuggaUugaagggagcuccuc	2	1	JCK-1
uuggaAugaagggagcuccuc	1	1	JCK-2
uuggGcugaagggagcuccuc	1	1	LT-3
uuggacugCagggagcuccuc	2	1	JCK-2
uuggacugaagggagcuUcuc	3	1	JCK-2
uuAgacugaagggagcuccuc	1	1	LT-3
uuggacugaagggagcuccuc	265	0	LCK-3
uNggacugaagggagcuccuc	1	1	JCK-3
uuggCcugaagggagcuccuc	3	1	JCK-2
uuggacugaagggagcucUuc	7	1	LT-1
uuggaUugaagggagcuccuc	3	1	JCK-3
uuggacugaagggagcuccuc	2	0	LCK-1
uuggacugaagggagcuccuc	1081	0	LT-1
uuggacugaagggaCcuccuc	1	1	JCK-3
uuggacugaagggagcuccuc	85	0	JT-2
uuggacugaagggagcucUuc	16	1	JCK-3
uuggacugGagggagcuccuc	1	1	JCK-2
uuggacugaCgggagcuccuc	1	1	LT-2
uuggCcugaagggagcuccuc	1	1	JT-3
uuggacugaaggUagcuccuc	1	1	JCK-2
uuggacugaaggAagcuccuc	1	1	JCK-1
uuggacugaCgggagcuccuc	2	1	LCK-3
Cuggacugaagggagcuccuc	1	1	LT-2
uuggacugaagggagcuccuc	1503	0	JCK-2

Star **Mature**

agguaauccaauggauaccaugaguggaagaguaauggaaagugagagcuuccuucagcccacucaugguaggauauaaaggggaugucgaaguaacugccgacucauucacucaagcacucaguagaagaaagggagauugguauuuugcuacugugauugugugaaugaagcgggagauauuuugcuucccucuuucucuaugcuuggacugaagggagcuccuccuuucauuugccucuauuauu

...........uuggacugaagggagcucUuc.....................	17	1	LT-2
...........uuggacugaagggagcucAuc.....................	1	1	LT-1
...........uuAgacugaagggagcuccuc.....................	1	1	JCK-1
...........uuUgacugaagggagcuccuc.....................	1	1	LT-2
...........uNggacugaagggagcuccuc.....................	3	1	JCK-1
...........uuggaUugaagggagcuccuc.....................	3	1	JCK-2
...........uuggacugaagggagcuccuc.....................	58	0	JT-1
...........uCggacugaagggagcuccuc.....................	1	1	JCK-2
...........uuggacugaagggagcuccuc.....................	271	0	LT-2
...........uuggCcugaagggagcuccuc.....................	2	1	JCK-1
...........uuggacugaagggagcGccuc.....................	2	1	JCK-2
...........uuggacugCagggagcuccuc.....................	1	1	JT-2
...........uuggacugCagggagcuccuc.....................	3	1	LT-2
...........uuAgacugaagggagcuccucc....................	1	1	JCK-3
...........uuggacugaagggagcuccucc....................	23	0	JCK-3
...........uuggacugaagggagcuccucU....................	10	1	LT-1
...........uuggacugaagggagcuccuUc....................	44	1	JCK-3
...........uuggacugaagggagcuccucA....................	1	1	LT-3
...........uuggacugaagggagcuccuUc....................	24	1	LT-1
...........uuggacugaagggagcuccucU....................	5	1	JCK-3
...........uuggacugaagggagcuccuUc....................	2	1	JT-2
...........uuggacugaagggagcuccucc....................	28	0	LT-3
...........uuggacugaagggagcuccuUc....................	29	1	LT-3
...........uuggacugaagggagcuccuUc....................	37	1	JCK-1
...........uuggacugaagggagcuccucc....................	11	0	LCK-3
...........uuggacugaagggagcuccuUc....................	3	1	LCK-2
...........uuggacugaagggagcuccucA....................	1	1	JCK-2
...........uuggacugaagggagcuccucU....................	3	1	LT-3
...........uuggacugaagggagcuccuUc....................	25	1	LCK-3
...........uuggacugaagggagcuccucc....................	34	0	JCK-2
...........uuggacugaagggagcuccuUc....................	13	1	JT-1
...........uuggacugaagggagcuccucc....................	7	0	JT-3
...........uuggacugaagggagcuccucc....................	22	0	LT-1
...........uuggacugaagggagcuccucU....................	9	1	JCK-2
...........uuggacugaagggagcuccuUc....................	53	1	JCK-2
...........uuggacugaagggagcuccucc....................	19	0	LT-2
...........uuggacugaagggagcuccucc....................	21	0	JCK-1
...........uuggacugaagggagcuccuUc....................	30	1	LT-2
...........uuggacugaagggagcuccuUc....................	2	1	JT-3
...........uuggacugaagggagcuccucA....................	1	1	LT-1
...........uuggacugaagggagcuccucU....................	2	1	JCK-1
...........uuggacugaagggagcuccucA....................	1	1	JCK-1
...........uuggacugaagggagcuccuccu...................	1	0	JCK-3
...........uuggacugaagggagcuccucUu...................	1	1	LT-1
...........uuggacugaagggagcuccCccu...................	9	1	LCK-3
...........uuggacugaagggagcuccuccu...................	1	0	JCK-1
...........uuggacugaagggagcuccuccA...................	1	1	LT-1
...........uuggacugaagggagcuccuccu...................	1	0	JCK-2
...........uuggacugaagggagcuccucUu...................	4	1	LT-2
...........uuggacugaagggagcuccuccu...................	3	0	LT-3
...........uuggacugaagggagcuccuccu...................	8	0	LT-2
...........uuggacugaagggagcuccCccu...................	3	1	LT-1
...........uuggacugaagggagcuccuccA...................	4	1	LT-2
...........uuggacugaagggagcuccuUcu...................	1	1	LT-2
...........uuggacugaagggagcuccuccu...................	7	0	LT-1
...........uuggacugaagggagcuccucUuu..................	3	1	LT-2
...........uuggacugaagggagcuccucUuu..................	1	1	JCK-3
...........uuggacugaagggagcuccuccCu..................	1	1	LT-1
...........uuggacugaagggagcuccucUuu..................	1	1	LT-1
...........uuggacugaagggagcuccucUuu..................	1	1	JT-2
...........uuggacugaagggagcuccuccGu..................	2	1	LT-2
...........uuggacugaagggagcuccucUuu..................	3	1	JT-3
...........uuggacugaagggagcuccucUuuu.................	1	1	LT-1
...........uuggacugaagggagcuccuccuuuC................	1	1	LT-1
...........uuggacugaagggagcuccuccuuuuc...............	1	0	JCK-3
...........uuggacugaagggagcuccuccuuuuc...............	1	0	JCK-2
...........uuggacugaagggagcuccuccuuuuc...............	3	0	JT-3
...........uuggacugaagggagcuccuccuuuuc...............	1	0	JCK-1
...........uuggacugaagggagcuccuccuuuuc...............	2	0	JT-2
............uggCcugaagggagcuccu......................	1	1	LT-2

Star Mature

```
agguaauccaauggauaccaugaguggaagaguaauggaaagugagagcuuccuucagcccacucauggauaggauauaaaggggaugucgaaguaacugccgacucauucacucaagcacucaguagaagaaagggagauugguauuuugcuacugugauugugugaaugaagcgggagauauuuugcuucccucuuucucuaugcuuggacugaagggagcuccuccuuuucauuugccucuauuauu
................................................................................................................................................................................................................uggacugaagggagcuGcu.......................	1	1	JCK-2
................................................................................................................................................................................................................ugUacugaagggagcuccu.......................	1	1	LT-1
................................................................................................................................................................................................................uggacugaagggagcuccuc......................	2	0	JCK-1
................................................................................................................................................................................................................uUgacugaagggagcuccuc......................	1	1	LT-1
................................................................................................................................................................................................................uggacugaagggagcuccuc......................	4	0	LT-1
................................................................................................................................................................................................................uggacugaagggagcuccuc......................	2	0	JCK-2
................................................................................................................................................................................................................uggacugaagggagcuccuc......................	4	0	JCK-3
................................................................................................................................................................................................................uggacugaagggagcuccuc......................	2	0	LT-2
................................................................................................................................................................................................................uggacugaagggagcuccucc.....................	3	0	JT-3
................................................................................................................................................................................................................uggacugaagggagcuccucc.....................	12	0	LT-3
................................................................................................................................................................................................................uggacugaagggagcuccuUc.....................	7	1	JT-2
................................................................................................................................................................................................................uggacugaagggagcuccuUc.....................	42	1	LCK-3
................................................................................................................................................................................................................uggacugaagggagcuccuUc.....................	36	1	JCK-3
................................................................................................................................................................................................................uggacugaagggagcuccuUc.....................	39	1	LT-3
................................................................................................................................................................................................................uggacugaagggagcuccucc.....................	3	0	JT-2
................................................................................................................................................................................................................uggacugaagggagcuccucc.....................	7	0	LT-2
................................................................................................................................................................................................................uggacugaagggagcuccuUc.....................	72	1	LT-1
................................................................................................................................................................................................................uggacugaagggagcuccuUc.....................	1	1	LCK-1
................................................................................................................................................................................................................uggacugaagggagcuccuUc.....................	244	1	LCK-2
................................................................................................................................................................................................................uggacugaagggagcuccucc.....................	11	0	LT-1
................................................................................................................................................................................................................uggacugaagggagcuccuUc.....................	80	1	JT-1
................................................................................................................................................................................................................uggacugaagggagcuccucc.....................	25	0	JCK-2
................................................................................................................................................................................................................uggacugaagggagcuccuUc.....................	3	1	LT-2
................................................................................................................................................................................................................uggacugaagggagcuccucc.....................	14	0	JCK-3
................................................................................................................................................................................................................uggacugaagggagcuccuUc.....................	79	1	JCK-2
................................................................................................................................................................................................................uggacugaagggagcucccCcc.....................	1	1	LT-1
................................................................................................................................................................................................................uggacugaagggagcuccuUc.....................	17	1	JT-3
................................................................................................................................................................................................................uggacugaagggagcuccuUc.....................	37	1	JCK-1
................................................................................................................................................................................................................uggacugaagggagcuccucc.....................	12	0	JCK-1
................................................................................................................................................................................................................uggacugaagggagcuccucc.....................	1	0	LCK-3
................................................................................................................................................................................................................uggacugaagggagcuccuccuuuuc................	1	0	JCK-3
.................................................................................................................................................................................................................ggacugaagggagcuccucc.....................	2	0	JCK-2
...................................................................................................................................................................................................................acugaagggagcuccucc.....................	2	0	LT-2
```
